# Invasive assessment of aortic stenosis in contemporary practice

**DOI:** 10.3389/fcvm.2022.1007139

**Published:** 2022-12-01

**Authors:** João Brito, Luís Raposo, Rui Campante Teles

**Affiliations:** ^1^Cardiovascular Intervention Unit, Hospital de Santa Cruz, Centro Hospitalar de Lisboa Ocidental, Lisbon, Portugal; ^2^Interventional Cardiology Center, Hospital da Luz, Lisbon, Portugal

**Keywords:** aortic stenosis, invasive assessment, echocardiography/catheterization discrepancies, pressure drop, low-flow, integrative approach

## Abstract

The authors review the current role of cardiac catheterization in the characterization of aortic stenosis, its main clinical applications, its pitfalls, and its additional value to the information provided by echocardiography. Discrepancies that may arise between these two modalities are discussed and further explained. Hemodynamic variables besides transvalvular pressure drop are described, and emphasis is given to an integrative approach to aortic stenosis assessment, that includes invasive and noninvasive evaluation.

## Introduction

Aortic valve stenosis (AS) is increasingly prevalent in developed countries, and its etiologies include congenital, degenerative (the most common), and rheumatic disease (1). Degenerative AS is a complex process of progressive inflammation, fibrosis, and calcification, affecting an otherwise structurally normal valve at the macroscopic level, which eventually leads to leaflet restriction and related hemodynamic consequences (2), and constitutes the main indication for aortic valve intervention. Regardless of etiology, stenosis of the aortic valve causes obstruction of the blood flow from the left ventricle (LV) to the aorta, which generates a systolic flow-dependent pressure drop (ΔP, a more accurate term for the widely used *gradient*) across the valve and chronic overload of the LV. Understanding the hemodynamic principles behind AS assessment allows us to critically integrate all the information provided by noninvasive diagnostic modalities and to acknowledge the important role of invasive hemodynamic studies in this setting.

## Essential anatomic and functional concepts underlying the measurements of aortic stenosis severity

**FIGURE 1:**
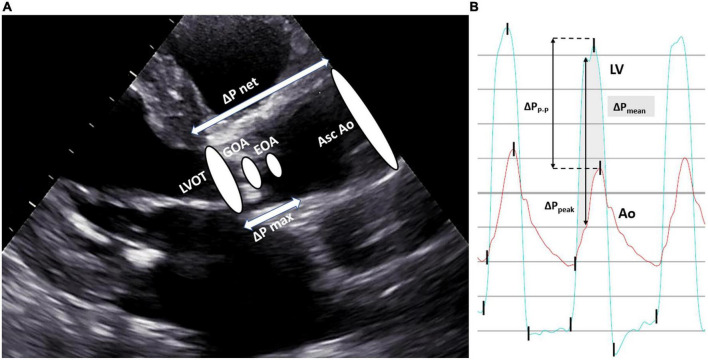
Echocardiographic and invasive characterization of aortic stenosis. Panel **(A)** The geometric orifice area (*GOA*) is the true anatomical area of the aortic valve and the area of the flow jet at the vena contracta, which occurs downstream of the valve orifice, is the effective orifice area (*EOA*), and corresponds to the calculated AVA by the continuity equation. GOA is always larger than EOA (they will be equal if GOA has the same size as LVOT). The pressure difference between the LVOT and EOA is known as ΔP_max_. The pressure difference between the ascending aorta and LVOT is ΔP_net_, as it is recorded after the occurrence of pressure recovery, and corresponds to the measured pressure drop in the catheterization laboratory. In the presence of the pressure recovery phenomenon, ΔP_max_ is higher than ΔP_net_, which partially explains the discrepancies between Doppler and invasive metrics. Panel **(B)** The shaded area represents the mean transaortic pressure drop (ΔP_mean_); peak-to-peak pressure drop (ΔP_P–P_) is the difference between the peak LV pressure and the peak aortic pressure at two different points in time; maximum instantaneous pressure drop (ΔP_peak_) is the maximum recorded difference between the LV and aortic pressure at the same point in time. *Ao indicates aortic pressure; Asc Ao, ascending aorta; LV, left ventricle pressure; LVOT, left ventricular outflow tract.*

In degenerative senile AS, calcification induces progressive leaflet immobility and obstruction, leading to a decrease in the aortic valve area (AVA). The narrowed AV orifice leads to the acceleration of blood through the valve, from a lower velocity in the LV outflow tract (LVOT) to the peak velocity at the vena contracta (VC) of the jet. The area between the free edges of the valve leaflets is the true anatomical measure of AVA and is known as the geometric orifice area (GOA). Although it can be measured by planimetry by using either computed tomography (CT) or echocardiogram (usually transesophageal echo), its assessment is challenging, namely due to dependence on image quality and difficulty to locating the exact plane of maximal leaflet opening in a tridimensional structure. The area of flow convergence at the VC is the echocardiographic-obtained AVA, i.e., the effective orifice area (EOA). The latter is smaller than the GOA and corresponds to the smallest measure of AVA. The pressure drop between the LVOT and the EOA is ΔP_max_. This decrease in pressure just distally to the valve, in the proximal ascending aorta, is primarily driven by the spatial acceleration of the blood flow (3). As the bloodstream flows to the distal ascending aorta, its kinetic energy is partially converted back into potential energy, resulting in an increase in local pressure. This phenomenon, known as the pressure recovery effect (Figure 1A), has implications for measurements and their interpretation, as will be further discussed below.

## Invasive and echocardiographic assessment of aortic stenosis severity

Current guidelines define severe AS as an AVA < 1.0 cm^2^ or indexed AVA (iAVA) < 0.6 cm^2^/m^2^, mean transvalvular pressure drop (ΔP_mean_) ≥ 40 mmHg, and/or peak transaortic velocity ≥ 4 m/s assessed by Doppler echocardiography (4). Indeed, AS is accurately diagnosed in a significant proportion of patients by Doppler echocardiographic assessment and this is mandatory to guarantee that only suitable patients are referred to valve intervention, considering that a faulty evaluation may prevent a patient from receiving the recommended treatment. In the past, invasive hemodynamic studies were critical for understanding the physiology and pathophysiology of valvular heart disease, but this role was downgraded with the advent of echocardiography, a noninvasive modality. Currently, cardiac catheterization for hemodynamic evaluation of AS is only recommended to accommodate any perceived inconsistencies between clinical and echocardiographic data or if non-invasive imaging is inconclusive (4).

It should be noted that a fundamental difference between these two techniques is that cardiac catheterization can directly measure actual pressure and pressure drops (ΔP), whereas Doppler ultrasound measures velocities that are converted into ΔP by applying the modified (and oversimplified) Bernoulli equation:ΔP=4*v*^2^, where *v* is the peak velocity measured by continuous Doppler through the LVOT and the aortic valve, in m/s. AVA can then be estimated from the velocities across the aortic valve and LVOT using the continuity equation:


$$A\,V\,A=\frac{L\,V\,O\,T\,a\,r\,e\,a\times L\,V\,O\,T\,V\,T\,I}{A\,V\,V\,T\,I}$$

where *VTI* is velocity time integral, measured by pulsed Doppler.

Although Doppler echocardiography has been established as the gold standard for assessing AS severity, it should be emphasized that echo parameters were initially derived as surrogates of invasive measurements and that there are important pitfalls that may jeopardize their accuracy. Echocardiography is highly operator-dependent, and image quality may be occasionally mediocre; a lack of alignment between the Doppler beam and the direction of the aortic jet can result in underestimation of the pressure drop and, on the other hand, the pressure drop may be overestimated in severe anemia or conditions associated with high output; AVA calculation relies on the accurate measurement of LVOT diameter, which is challenging and prone to intraobserver and interobserver variability (ranging from 5 to 8%) (5). Since the square of the radius is used to derive the area of the LVOT in the continuity equation, a small measurement error causes a significant error in AVA. Moreover, the LVOT shape is elliptical rather than circular in most of the patients, which may further result in underestimation or overestimation of echo-derived areas (5). Finally, the use of the simplified Bernoulli formulation may introduce a variable source of error, as further discussed in the following section.

For the assessment of AS severity in the cardiac catheterization laboratory, it is essential to accurately measure both the transvalvular pressure drop and cardiac output (CO; flow). ΔP can be obtained by simultaneous measurement of LV and ascending aorta pressures, either by using two arterial accesses, dual lumen fluid-filled catheters, multitransducer micromanometer catheters, or common pressure wires (PWs) (6). Care must be taken with the potential damping of aortic pressure with double-lumen catheters, which may falsely increase the pressure drop. Also, the cross-sectional area of a catheter crossing the aortic valve may increase the measured pressure drop, especially in very tight stenoses, and there is *in vitro* evidence that catheter geometry may produce significant measurement bias in both the peak pressure and the waveform shape (7). In our experience, 4-to-5 French catheters (pigtail or multipurpose shapes with side holes) will be adequate for most cases. The use of a PW in the LV for pressure measurement further obviates these issues, however, at the expense of a higher procedural cost and the possible need for post-procedure analysis, as some polygraphs will not co-register both PW and fluid-filled signals simultaneously. Non-disposable multitransducer micromanometer catheters are very accurate but costly and are less often used in clinical laboratories (6). Catheterization should allow for the measurement of both ΔP_P–P_, i.e., the peak-to-peak systolic pressure drop (the difference between the peak LV pressure and the peak aortic pressure), and ΔP_mean_, i.e., the invasive mean pressure drop (average of instantaneous pressure drops over the ejection period) (Figure 1B). It is important to emphasize that single-catheter pullback curves from the LV to the aorta provide an approximation of the peak-to-peak systolic pressure drop, which is not a physiological measurement since it is obtained at two different points in time and, as such, may be imprecise for diagnostic purposes. The mean pressure drop should be used for diagnosis and measured from at least 3 consecutive beats in patients with sinus rhythm or 8–10 consecutive beats when a rhythm is irregular (8). CO is usually assessed invasively by the Fick method or thermodilution. The Fick method is the gold standard and requires the measurement of real-time oxygen consumption using dedicated equipment, which can be time-consuming and unpractical in the catheterization laboratory. Alternatively, oxygen consumption may be estimated from gender- and age-specific nomograms (indirect Fick method), which constitutes a potential source of error, as the impact of disease states is not accounted for. When thermodilution is used, inaccuracy may result from severe tricuspid regurgitation, cardiac shunts, very low output states, and highly irregular rhythms (8).

Finally, AVA can be calculated from ΔP and CO using the Gorlin equation (9):


$$A\,V\,A\,\left(c\,m^{2}\right)=\frac{\mathrm{CO}\,\left(l/\min\right)/\left[\mathrm{HR}\,\left(\text{bpm}\right)\,x\,\mathrm{SEP}\,\left(\text{mSec}\right)\right]}{44.3\,x\,\sqrt{\Delta \,\text{P}}\,\left(m\,m\,H\,g\right)}$$

where SEP is the systolic ejection period, K = 44.3 (empirical constant), and ΔP is the mean pressure drop. It must be noted that this equation has several inherent limitations (mainly stemming from the fact that it has not been primarily derived for the aortic valve) and that accuracy may be lower in patients with bradycardia, tachycardia, aortic regurgitation, or low output states (10).

Thorough invasive evaluation of a patient with AS is multiparametric. It must include measurement of transvalvular pressure drop, CO, and calculation of AVA, but also an appraisal of left ventricular contractility and peripheral vascular resistance. In addition, other indexes can be used to arbitrate inconsistency. Aortic valve resistance can be easily calculated using the same essential parameters and has been suggested to be less flow-dependent than the Gorlin-derived AVA (11). In the end, critical interpretation and integration of all the obtained values are mandatory for a correct diagnosis.

Questions have been raised regarding the risk of embolic stroke resulting from aortic root manipulation and retrograde aortic valve crossing, with one study showing a high frequency of magnetic resonance imaging (MRI) defects in this context (12). However, a subsequent study failed to corroborate these findings (13). One further study showed that the time required for crossing the aortic valve was the most important independent predictor of silent cerebral infarction (14). In this context, the increasing number of transcatheter aortic valve interventions (TAVI) has brought technical and device improvements which have reduced the procedure time and increased its efficacy and safety (15). Besides this potential issue, one should also notice other known complications of cardiac catheterization, such as local vascular injury, bleeding complications, and also the exposure of both patients and operators to ionizing radiation. Taking all these aspects into consideration, the decision for invasive assessment of AS should be judiciously made, when a gain of diagnostic ability is expected, as unnecessary cardiac surgery and TAVI are themselves associated with a risk of neurologic and other systemic complications (16, 17). It is wise to recommend that this procedure should be performed by experienced operators.

## Discrepancies between echocardiographic and catheterization findings

There is evidence that the correlation between noninvasive and invasive AS assessments is weaker than previously reported (18, 19). This observation should be highlighted and critically appraised since the values used in guidelines to define severe AS are derived from outcome studies using invasive hemodynamics, whereas echo values are recommended to evaluate AS (20). As previously discussed, several sources of error exist in both echocardiographic and invasive evaluation that can contribute to this discrepancy, starting with the essential assumptions inherent to both techniques. Transvalvular pressure drops derived from catheterization are lower than echo-derived values, and traditionally, this observation has been mainly explained by the pressure recovery phenomenon (21, 22). While Doppler echocardiography measures ΔP from the velocity obtained at the *VC*, catheterization directly measures the pressure drop between LVOT and the ascending aorta, after the conversion of some kinetic energy back into potential energy (ΔP_net_) (Figure 1A). The degree of pressure recovery depends on many factors, including the ratio of actual AVA/ascending aorta area, with more pressure recovery typically observed in patients with larger valve orifice and smaller ascending aorta (22). Therefore, in the presence of significant pressure recovery, invasive pressure drops are lower and the estimated AVA is higher than the corresponding echocardiographic values. However, the exact anatomic point where the pressure is fully recovered is not known and differs from subject to subject, which potentially introduces a source of error in invasive estimations.

Moreover, the very use of the modified Bernoulli equation for the noninvasive assessment of ΔP provides an additional and important explanation for these discrepancies. It relies on two assumptions: (1) the pressure drop is entirely due to spatial acceleration of blood flow, neglecting the impact of unsteady and viscous components, and (2) the blood flow is considered a single streamline, which neglects the velocity distribution across the aortic valve plane (3). While it is known that the first assumption is indeed accurate as the spatial acceleration of blood is the dominant pressure component, there is evidence that the use of a single peak velocity value to the detriment of a complete velocity profile results in consistent overestimation of transvalvular pressure drop and is a source of uncontrolled variability (3, 23).

A comparison of the most important features of these two modalities for AS severity assessment is summarized in Table 1.

**TABLE 1:** Comparison of cardiac catheterization and Doppler echocardiography for AS evaluation.

Modality	Direct measurements	Advantages	Pitfalls
Cardiac catheterization	Mean transaortic pressure drop (ΔP_mean)_ Maximum instantaneous transaortic pressure drop (ΔP_peak)_ Peak-to-peak transaortic pressure drop (ΔP_P–P_) Cardiac Output	Direct pressure measurement	Invasive Radiation exposure Potential risk of embolic stroke Unknown exact anatomic point where full pressure recovery occurs
Doppler echocardiography	Instantaneous VC velocity Peak VC velocity (V_max_) Instantaneous transaortic pressure drop (through modified Bernoulli equation) Mean transvalvular pressure drop (Doppler ΔP_mean)_	Noninvasive Anatomic Evaluation Widely accessible	Requires good imaging window Does not provide pressure directly LVOT measurement may decrease AVA calculation accuracy Assumption of a single peak velocity value in Bernoulli equation may overestimate the pressure drop

*AS indicates aortic stenosis; AVA, aortic valve area; LVOT, left ventricular outflow tract; VC, vena contracta.*

## Role of cardiac catheterization in low-flow states

### Low-flow, low-gradient aortic valve stenosis

A subset of patients with severe AS presents with low CO, ΔP_mean_< 40 mmHg, and reduced LV ejection fraction. The challenge in this setting is to ensure that the small, calculated AVA is due to true severe AS or “pseudo-aortic stenosis”. In the latter, the aortic valve has the moderate disease, but the leaflet opening is insufficient due to a weak LV, with reduced inotropy. As the Gorlin formula is flow-dependent, the severity of AS may be overestimated in this situation. Dobutamine infusion (whether during catheterization or echocardiography) is the gold standard to differentiate true AS from pseudostenosis, as it induces an increase in inotropy and, consequently, increases CO. In true AS, ΔP_mean_ rises to ≥ 40 mmHg. If CO normalizes but ΔP_mean_ remains low (< 30 mmHg), with an increase in AVA, then pseudostenosis is present. Some patients will not be able to increase CO due to a lack of contractile reserve (defined as an increase in stroke volume <20%). These subjects have indeterminate AS and will require the integration of clinical, imaging, and laboratory parameters for a comprehensive evaluation, bearing in mind that the long-term prognosis after valve intervention is poorer in this group of patients (24).

### Paradoxical low-flow, low-gradient aortic valve stenosis

This group of patients presents with iAVA < 0.6 cm^2^/m^2^, ΔP_mean_< 40 mmHg, LVEF > 50%, and indexed stroke volume (SVI) < 35 ml/m^2^. Despite the preserved ejection fraction, the low-flow state may generally be explained by higher LV filling pressures, reduced systolic longitudinal myocardial shortening, and increased afterload on the LV through decreased systemic arterial compliance and increased systemic vascular resistance causing higher vascular impedance. This pattern may lead to an underestimation of AS severity and prevent appropriate valve intervention. Cardiac catheterization has an important role in the evaluation of this entity when noninvasive metrics are inconclusive or if there is a discrepancy between clinical and echocardiographic findings. One approach in this setting is to evaluate the global LV hemodynamic burden by determining the valvulo-arterial impedance (Z_va_) by the following equation (25):


$$Z\,v\,a=\frac{\text{SBP}+\Delta \,\text{Pmean}}{\text{SVI}}$$

where SBP is systolic blood pressure and SVI is indexed stroke volume.

Patients with paradoxical low-flow, low-gradient AS tend to have Z_va_ ≥ 5.5 mmHg/ml/m^2^, and these values are associated with a worse prognosis (26). In this setting, a nitroprusside challenge may be further helpful to assess the fixed component of total left ventricular afterload, unmask true aortic stenosis (27), and ideally predict symptomatic response to hemodynamic relief of aortic stenosis.

## Clinical application of invasive assessment of Aortic valve stenosis

To illustrate the previously outlined concepts, we present the case of an 81-year-old overweight woman with a history of chronic obstructive lung disease, deep vein and pulmonary thromboembolism, and reactive depression, who was evaluated in the outpatient clinic with complaints of fatigue and effort dyspnea (New York Heart Association class II). The echocardiogram showed preserved ejection fraction (LVEF 57%) and calcified AS, with a mean pressure drop of 26 mmHg, AVA 0.36 cm^2^/m^2^, and SVI 26 ml/m^2^. These findings were consistent with the diagnosis of paradoxical low-flow, low-gradient AS. However, doubts persisted regarding the severity of AS, and the presence of significant comorbidities suggested that the functional limitation might be due to other causes. Further investigation by CT scan revealed an aortic valve calcium score of 569 AU, which suggested that severe AS was less likely (28). An invasive hemodynamic assessment was performed to reconcile these discrepancies: ΔP_mean_ was 20 mmHg, CO was 5.1 L/min, and SVI was 32.1 ml/m^2^. AVA estimated by the Gorlin equation was 1.16 cm^2^. Zva was not elevated (4.4 mmHg.ml^–1^.m^2^) as would be expected in paradoxical low-flow low-gradient AS and the intrinsic valve resistance was < 120 dynes.s.cm^–5^. The usefulness of this latter variable remains controversial, although one study postulates that in low-flow, low-gradient aortic stenosis, such a value identifies pseudo stenosis, while the values of > 180 dynes.s.cm^–5^ identify truly severe AS (11). Integration of all these invasive parameters allowed us to exclude severe AS and reclassify it as moderate.

## Conclusion and future perspectives

Currently, invasive hemodynamic evaluation of AS is indicated to clarify inconsistencies between clinical and echocardiographic findings, or when those findings are not conclusive. Although AS severity assessment relies mostly on the echocardiographic evaluation, one should be aware that the obtained metrics often differ from invasive parameters. While catheterization allows for direct measurement of pressure drop, Doppler echocardiography measures velocities that are converted into pressure drops. This is an essential and distinguishing feature between these two modalities that must be accounted for when interpreting the whole clinical picture. It is critical to understand the hemodynamic concepts behind AS evaluation to identify potential inconsistencies in diagnosis and the subsets of patients that benefit from an integrated approach that includes cardiac catheterization.

Also, although AS is a valve disease, looking exclusively at the valve may be deceiving and it should be noted that understanding the coupling between the valve and the LV is equally essential. Thus, investigation of the extent of myocardial fibrosis (by using cardiovascular magnetic resonance (CMR), echocardiography, or quantification of brain natriuretic peptide levels) may be useful in determining the prognostic impact of AS and potential valve intervention (29, 30). Recent advances in CMR have also proven to provide more precise and accurate values of pressure drop by addressing the limitations of the simplified Bernoulli formulation (3, 31). Interestingly, imaging modalities such as 4D flow CMR have the advantage of acquiring three-dimensional blood velocity vector fields, which have been validated against gold-standard techniques, and potentially overcome a previously discussed limitation of Doppler echocardiography (31). Unanswered questions, whether 4D flow CMR-derived pressure computations correlate accurately with transduced pressure data and whether these measures prove to have a strong prognostic impact, are a field of promising current and future research.

Finally, AS has increasingly become a disease of the elderly and is likely accompanied by multiple comorbidities including LV dysfunction, coronary artery disease, lung disease, and frailty. As such, their symptoms might arise from causes other than aortic stenosis, and establishing this link is often complicated and ambiguous. While cardiac catheterization has a role in clarifying AS severity, a noninvasive test such as a cardiopulmonary exercise test may help to define the symptomatic status and the functional capacity of such patients.

In the current era of expanding TAVI, we have observed a trend for an increased referral of AS patients with different degrees of severity and different hemodynamic states. The role of cardiac catheterization in the accurate hemodynamic characterization of the disease should be considered at a lower threshold, and this integrated, multimodality approach should become the cornerstone of patients’ evaluation for treatment decisions and the best counseling.

## Author contributions

JB wrote the first draft of the manuscript. JB, LR, and RT wrote sections of the manuscript. All authors contributed to manuscript revision, read, and approved the submitted version.
